# Association of screen exposure/sedentary behavior and precocious puberty/early puberty

**DOI:** 10.3389/fped.2024.1447372

**Published:** 2024-09-23

**Authors:** Xinyu Wu, Lingmei Wang, Peng Xue, Jingyi Tang, Haodong Wang, Huijun Kong, Cuilan Lin, Bo Chang, Shijian Liu

**Affiliations:** ^1^ Department of Endocrinology, Dalian Women and Children’s Medical Group, Dalian Medical University, Dalian, China; ^2^ Department of Pediatrics, The People’s Hospital of Suzhou New District, Suzhou, China; ^3^ Department of Clinical Epidemiology and Medical Statistics, Pediatric Translational Medicine Institute, Shanghai Children’s Medical Center, School of Medicine, Shanghai Jiao Tong University, Shanghai, China; ^4^ Department of Epidemiology and Statistics, School of Public Health, School of Medicine, Shanghai Jiao Tong University, Shanghai, China; ^5^ Department of Pediatrics, Qufu People’s Hospital, Qufu, China; ^6^ Department of Children Health Care, Boai Hospital of Zhongshan, Southern Medical University, Zhongshan, Guangdong, China

**Keywords:** screen exposure, sedentary behavior, precocious puberty, early puberty, obesity

## Abstract

**Background:**

In recent years, with the development of society, children's daily exposure to screen time has gradually increased. Screen exposure and sedentary behavior have brought a host of harms to children's lives. The aim of this study was to explore the effects of screen exposure and sedentary behavior on precocious puberty and early development.

**Methods:**

This is a cross-sectional study in the school-based population. A total of 3,560 children were recruited from Qufu City, Shandong province using multistage stratified cluster random sampling. All study subjects had a physical examination by professional pediatricians in October 2019, and were investigated with health questionnaires. Precocious puberty is defined as development of secondary sexual signs in boys before 9 years or in girls before 8 years. Screen time was calculated as the average of screen time on weekdays and weekend days, and sedentary time was calculated as the average of sedentary time on weekdays and weekend days. After adjusting for potential confounders, logistic regression was used to examine the association between screen exposure and sedentary behavior and early puberty and precocious puberty.

**Results:**

Sedentary time was a risk factor for precocious puberty and early development (OR = 1.428, 95% CI = 1.087–1.876) in girls without adjustment. No significant association was found between screen exposure and early puberty and early development both in girls and boys.

**Conclusions:**

Excessive sedentary behavior was associated with an increased risk of early puberty, especially in girls, while there was no significant association between screen exposure and early puberty and early development. In addition, further longitudinal investigations are needed to determine the causal relationship between screen exposure, sedentary behavior and precocious puberty.

## 1 Introduction

Precocious puberty (PP) is a common endocrine disease in children. Precocious puberty has been shown to expedite skeletal maturation in children, resulting in short stature (1, 2). Additionally, it has been associated with an elevated risk of stroke, type 2 diabetes, estrogen-dependent cancer, and other diseases (3, 4). Several studies have also established a relationship between precocious puberty and more dangerous behaviors or low self-esteem (5). It is noteworthy that the global prevalence and incidence of precocious puberty is on the rise, accompanied by an earlier onset age. Notably, the crude prevalence rates of precocious puberty in Taiwanese children exhibited a substantial increase in 2013 compared to 2,000 (boys from 0.99/10,000 to 7.01/10,000, and girls from 13.56/10,000 to 110.95/10,000) (6). Study based on the Korean National Insurance Registry showed that from 2008 to 2014, the overall incidence of central precocious puberty (CPP) was 122.8 per 100,000 individuals (262.8 per 100,000 for girls; 7.0 per 100,000 for boys). Among girls under 9 years, the incidence of CPP increased by 4.7 times, while among boys under 10 years, the incidence of CPP increased by 9.2 times (7). From 1998 to 2017, the incidence of CPP rose from 2.6 per 10,000 to 14.6 per 10,000, representing a 15-fold increase among Danish boys (8). Researchers in Copenhagen discovered that in 1991 and 2006, the age of menarche for girls was significantly earlier, with values of 13.42 and 13.13, respectively (9). The findings of a meta-analysis revealed a significant decline in the age of breast development among girls from 1977 to 2013, with a decrease of 0.24 years every decade. Additionally, there was a global trend of early onset in child sexual development, prompting the medical community to reconsider the definition of PP (10). In early 2023, China introduced the most recent iteration of the Expert Consensus on the Diagnosis and Treatment of CPP (2022), which revised the diagnostic criteria for PP in girls to include breast development before 7.5 years or menarche before 10.0 years (11).

In the past two to three decades, there has been a notable rise in the amount of time that children spend engaging with screen devices (12, 13). While the introduction of newer interactive screen devices may offer certain advantages, excessive utilization of these devices has been linked to potential cognitive and socio-emotional disorders, diminished physical activity, increased energy intake, reduced sleep duration, sleep disturbances, and the risk of obesity (14, 15). Furthermore, research has demonstrated that environmental endocrine disruptors (EEDs), genetic factors, early maternal menarche, intrauterine growth retardation, elevated body mass index (BMI), and obesity exert a significant influence in early puberty (16, 17). Hence, we hypothesized that in this study, prolonged screen exposure during childhood could potentially contribute to the onset of early puberty, with obesity potentially acting as a mediating factor.

To the best of our knowledge, there are no research investigating the association between screen exposure, sedentary behavior, and pubertal development. In order to address this gap, we conducted a cross-sectional investigation in a Chinese city, aiming to examine the impact of screen exposure and sedentary behavior on the incidence of precocious puberty and early puberty.

## 2 Methods

### 2.1 Study subjects and study design

This cross-sectional study was conducted among a school-based population and was the baseline survey for a prospective cohort study. Prior to their participation in the study, all guardians provided written informed consent. The study received approval from the Ethics Committees of Shanghai Children's Medical Center, and Qufu People's Hospital. The research was conducted in Qufu City in Shandong Province. From October 11 to December 5, 2019, a multi-stage stratified cluster random sampling approach was employed to select children in grades 1–3, aged 5–10 years. During this period, physical examinations were conducted, and general demographic characteristics of the participants were collected, including age, gender, residence, family income, parental education, screen time, sedentary time, sleep time, and physical activity. This study included a total of 3,560 children, The recruitment process was described in our previous study (18).

### 2.2 Assessment and definitions of screen exposure and sedentary behavior

Screen time or screen exposure refers to the duration individuals spend engaging with electronic or digital media, such as television, tablets, or computers. Sedentary behavior, on the other hand, pertains to any energy expenditure below 1.5 Metabolic equivalent (MET)while in a seated, leaning, or awake lying position (19). In this study, sedentary behavior encompasses sitting in various settings including school, home, and outdoors, as well as sitting while engaged in activities such as desk work, socializing, reading, or watching television. The study further categorizes screen exposure and sedentary behavior into two distinct contexts: weekdays and weekends, ultimately examining the duration of screen time. The screen time finally included is the average of working days and weekend ((screen time on working days* 5 + screen time on weekend days * 2)/7). In this study, the Q1 (first quartile), Q2 (second quartile), Q3 (third quartile) of screen time were 0.6 h, 1.1 h and 2.0 h, respectively. Sedentary time is the average of working days and weekend days [(sedentary time on working days* 5 + sedentary time on weekend days*2)/7]. In this study, the Q1, Q2, Q3 of sedentary time were 1.1 h, 2.7 h and 5.4 h, respectively. According to the guidelines of physical activity for Chinese children and adolescents, it is believed that Chinese children and adolescents should limit screen time or sedentary behavior to no more than 2 h per day.

### 2.3 Evaluation and definitions of precocious puberty/early development

All enrolled students underwent individual physical examinations conducted by endocrinologists or pediatricians. Female participants received breast development and pubic hair assessments through examination and palpation performed by a female pediatrician in a private setting. In the case of overweight or obese girls, the diagnosis of precocious puberty was determined using Tanner staging in conjunction with ultrasound examination. Male participants had their testicular volume measured by male physicians through palpation and compared to an orchidometer. Pubic hair development was evaluated through visual inspection for both genders. The assessment of breast, pubic hair, and testicular development during pubertal stages was conducted using the Tanner staging method, as outlined in the Guidelines for the Diagnosis and Treatment of Precocious Puberty released by the Ministry of Health of China in 2010 (20). According to these guidelines, precocious puberty in girls is characterized by breast or pubic hair development reaching stage II or above before the age of 8, or menarche occurring before the age of 10. In boys, precocious puberty is defined as pubic hair or testicular development reaching stage II or above before the age of 9. Since there is currently no standard definition of early development, we used the definition most commonly used in other studies to increase the comparability of our findings. The definition of early puberty was younger than the median age in each of the pubertal Tanner stages (II, III, IV, and V). The median age in each pubertal development stage comes from a multi-center population study in China, and the data are representative to Chinese children to a certain extent (21, 22).

### 2.4 Statistical analysis

Categorical variables were presented as percentages and frequencies (%), and statistical differences were assessed using the Pearson chi-square test. Logistic regression was employed for multiple analyses, with odds ratios (OR) and 95% confidence intervals (CIs) utilized. The data were analyzed using IBM SPSS Statistics 25.0, employing a two-sided analysis, and a *P*-value < 0.05 was considered statistically significant.

## 3 Results

### 3.1 Basic characteristics of the study population

Table 1 displays the characteristics of children categorized by gender and pubertal status, encompassing 3,238 (91.0%) children with normal puberty, 206 (6.3%) children with early puberty, and 116 (3.3%) children with precocious puberty. In the PP group, there was a higher percentage of girls (74.1%) compared to boys (25.9%). Both boys and girls with overweight or obesity, who experiencing precocious puberty or early puberty are more than those with normal weight. For girls, in addition to BMI, factors influencing precocious puberty or early puberty also include sedentary time and the father's education level.

**Table 1:** Characteristics for participants according to sex and puberty stage.

Variables	Normal *N* = 3,238	Early puberty *N* = 206	Precocious puberty *N* = 116	*χ* ^2^	*P*
Boys					
Screen time (h/day)					** **
<2	1,371 (73.6)	9 (56.3)	23 (76.7)	2.616	0.270
≥2	491 (26.4)	7 (43.8)	7 (23.3)		
Sedentary time (h/day)					
<2	270 (14.5)	1 (6.3)	4 (13.3)	0.503	0.791
≥2	1,592 (85.5)	15 (93.8)	26 (86.7)		
BMI					
Normal	1,037 (55.7)	6 (37.5)	7 (23.3)	19.785	**<0**.**001**
Skinniness	148 (7.9)	0 (0.0)	1 (3.3)		
Overweight/obesity	677 (36.4)	10 (62.5)	22 (73.3)		
MVPA(min/day)				* *	* *
<60	1,813 (97.4)	15 (93.8)	30 (100.0)	1.621	0.469
≥60	49 (2.6)	1 (6.3)	0 (0.0)		
Sleep duration(h/day)					
<9	153 (8.2)	3 (18.8)	3 (10.0)	8.689	0.056
9–10	845 (45.4)	7 (43.8)	18 (60.0)		
>10	864 (46.4)	6 (37.5)	9 (30.0)		
Mother's education level					
Low	592 (31.8)	4 (25.0)	12 (40.0)	2.860	0.582
Middle	690 (37.1)	6 (37.5)	7 (23.3)		
High	580 (31.1)	6 (37.5)	11 (36.7)		
Father's education level					
Low	441 (23.7)	4 (25.0)	7 (23.3)	0.455	0.978
Middle	728 (39.1)	5 (31.3)	12 (40.0)		
High	693 (37.2)	7 (43.8)	11 (36.7)		
Total family income					
Low	662 (35.6)	6 (37.5)	5 (16.7)	5.892	0.187
Middle	955 (51.3)	7 (43.8)	20 (66.7)		
High	245 (13.2)	3 (18.8)	5 (16.7)		
Girls					
Screen time (h/day)					
<2	1,031 (74.9)	139 (73.2)	61 (70.9)	0.889	0.641
≥2	345 (25.1)	51 (26.8)	25 (29.1)		
Sedentary time (h/day)					
<2	989 (71.9)	131 (68.9)	46 (53.5)	13.454	**0**.**001**
≥2	387 (28.1)	59 (31.1)	40 (46.5)		
BMI					
Normal	941 (68.4)	96 (50.5)	27 (31.4)	147.982	**<0**.**001**
Skinniness	180 (13.1)	7 (3.7)	1 (1.2)		
Overweight/obesity	255 (18.5)	87 (45.8)	58 (67.4)		
MVPA(min/day)					
<60	1,359 (98.8)	188 (98.9)	85 (98.8)	0.048	0.976
≥60	17 (1.2)	2 (1.1)	1 (1.2)		
Sleep duration(h/day)					
<9	80 (5.8)	20 (10.5)	4 (4.7)	8.205	0.084
9–10	613 (44.5)	89 (46.8)	40 (46.5)		
>10	683 (49.6)	81 (42.6)	42 (48.8)		
Mother's education level					
Low	419 (30.5)	64 (33.7)	29 (33.7)	5.493	0.240
Middle	478 (34.7)	74 (38.9)	25 (29.1)		
High	479 (34.8)	52 (27.4)	32 (37.2)		
Father's education level					
Low	319 (23.2)	57 (30.0)	25 (29.1)	10.863	**0**.**028**
Middle	495 (36.0)	77 (40.5)	28 (32.6)		
High	562 (40.8)	56 (29.5)	33 (38.4)		
Total family income					
Low	466(33.9)	72(37.9)	22(25.6)	5.097	0.277
Middle	716(52.0)	92(48.4)	47(54.7)		
High	194(14.1)	26(13.7)	17(19.8)		

BMI, body mass index; WHtR, waist-to-height ratio; MVPA, moderate-to-vigorous physical activity. Screen time is the mean of video viewing time, VG time and Oline time on weekdays and day off. Sedentary time is the mean of sedentary time on weekdays and day off. Sleep duration is the sum of sleep duration during day and night on weekdays and day off. Total family income (low: <50,000 Chinese yuan per year; middle: 50,000–150,000 Chinese yuan per year; and high: ≥150,000 Chinese yuan per year). Parental education (low: middle school or below; middle: high school or technical school and high: college degree or more). Overweight and obesity were classified according to the international (IOTF) childhood BMI cut-offs.

*P* < 0.05 was marked in bold.

### 3.2 Relationship between screen time and puberty by ages and gender

Table 2 presents the relationship between screen time and pubertal stage across different ages and genders. The boys were divided into three groups based on age (Age <8, 8≤ Age <9, Age ≥9), with the age group <8 having the largest number of cases (685) and no significant correlation between screen time and puberty observed in any of the three age groups (*P *> 0.05). Similarly, the girls were divided into three groups (Age <7, 7 ≤Age <8, Age ≥8), with the Age 8 group had the largest number (860 cases), there was no significant correlation between screen time and puberty observed in any of the three age groups (*P *> 0.05).

**Table 2:** The association between screen time and pubertal stage according to sex and age.

Variables	Normal *N* = 3,238	Early puberty *N* = 206	Precocious puberty *N* = 116	χ^2^	*P*
Boys					
Age <8					
Screen time <2	822 (74.9)	0 (0.0)	5 (83.3)	0.225	0.702
Screen time ≥2	275 (25.1)	0 (0.0)	1 (16.7)		
8 ≤ Age <9					
Screen time <2	471 (71.3)	0 (0.0)	18 (75.0)	0.159	0.820
Screen time ≥2	190 (28.7)	0 (0.0)	6 (25.0)		
Age ≥9					
Screen time <2	78 (75.0)	9 (56.3)	0 (0.0)	2.445	0.138
Screen time ≥2	26 (25.0)	7 (43.8)	0 (0.0)		
Girls					
Age <7					
Screen time <2	298 (83.9)	0 (0.0)	16 (80.0)	0.216	0.548
Screen time ≥2	57 (16.1)	0 (0.0)	4 (20.0)		
7 ≤ Age <8					
Screen time <2	399 (75.6)	0 (0.0)	43 (67.2)	2.119	0.145
Screen time ≥2	129 (24.4)	0 (0.0)	21 (32.8)		
Age ≥8					
Screen time <2	334 (67.7)	139 (73.2)	2 (100.0)	2.351	0.285
Screen time ≥2	159(32.3)	51(26.8)	0(0.0)		

### 3.3 Relationship between screen or sedentary time and early or precocious puberty

The logistic regression analysis presented in Table 3 examined the correlation between screen time and early puberty. To account for potential confounding factors, such as age, BMI, moderate-to-vigorous physical activity (MVPA), sleep time, parental education, and total family income, logistic regression was employed to investigate the association between screen exposure time, sedentary time, and precocious puberty in relation to early development. None of the models demonstrated statistically significant differences in the relationship between screen time and precocious puberty in relation to early development (*P *> 0.05). In the case of females, the unadjusted regression model (model 1) demonstrated that sedentary time was associated with an increased risk of early puberty and early development (OR = 1.428, 95% CI = 1.087–1.876). But this association disappeared after controlling for age, BMI, MVPA, sleep time, parental education, and total family income in subsequent models (model 2, model 3, and model 4). Conversely, we did not observe an association between sedentary time, and precocious puberty in relation to early development in the male population (*P *> 0.05).

**Table 3:** The regression of screen time and sedentary time on early puberty and precocious puberty according to sex.

Variables	Model 1		Model 2		Model3		Model4	
	OR (95% CI)	*P*	OR (95% CI)	*P*	OR (95% CI)	*P*	OR (95% CI)	*P*
Boys								
Screen time(h/day)								
<2	1.00	0.550	1.00	0.698	1.00	0.726	1.00	0.581
≥2	1.214 (0.642,2.295)		1.139 (0.590,2.199)		1.125 (0.582,2.177)		1.211 (0.614,2.388)	
Sedentary time(h/day)								
<2	1.00	0.499	1.00	0.441	1.00	0.497	1.00	0.686
≥2	1.381 (0.541,3.528)		1.471 (0.551,3.930)		1.412 (0.522,3.817)		1.230 (0.450,3.360)	
Girls								
Screen time(h/day)								
<2	1.00	0.405	1.00	0.600	1.00	0.578	1.00	0.525
≥2	1.132 (0.846,1.515)	** **	0.917 (0.664,1.268)		0.911 (0.658,1.263)		0.898 (0.646,1.249)	
Sedentary time(h/day)		** **						
<2	1.00	**0**.**010**	1.00	0.075	1.00	0.087	1.00	0.083
≥2	1.428 (1.087, 1.876)		1.315 (0.973,1.778)		1.304 (0.963,1.766)		1.310 (0.965,1.778)	

Model 1: no adjustment. Model 2: adjusted for age and BMI. Model 3: further adjusted for MVPA and sleep duration. Model 4: further adjusted for parents’ education level and total family income.

*P* < 0.05 was marked in bold.

## 4 Discussion

### 4.1 Summary and overview of the main findings

This research constitutes a population-based cross-sectional study, which has yielded findings indicating a correlation between sedentary behavior and early or precocious puberty. Specifically, for girls, the study found noteworthy positive correlations between sedentary behavior and early or precocious puberty. However, this correlation disappeared after adjusting for potential confounding variables such as age, BMI, MVPA, sleep duration, parental education, and family income. Conversely, no significant association between sedentary behavior and precocious or early puberty was observed in boys.

### 4.2 Possible mechanism of precocious puberty due to sedentary behavior and screen exposure

Sedentary behavior can be divided into two types: Sitting without looking at the screen and sitting in front of the screen; the latter is called screen time (23).

Melatonin, a hormone secreted by the pineal gland, is implicated in the regulation of the hypothalamic-pituitary-gonadal (PHG) axis (24). Melatonin controls the secretion of luteinizing hormone (LH) and follicle-stimulating hormone (FSH) by periodically down-regulating the expression of gonadotropin-releasing hormone (GnRH) gene, and thus has physiological effects on reproductive and sexual maturity of mammals (25). Furthermore, melatonin secretion exhibits a distinct circadian rhythm, with suppression during daylight hours and activation during the night. The synthesis of melatonin is dependent on light exposure, including its duration and frequency. The occurrence of precocious puberty may be influenced by the intensity of light (26). Light signals are mediated by melatonin to the hypothalamic-pituitary-gonadal axis (HPGA) to regulate sexual maturation and reproductive activities, prolonged and increased screen exposure, as well as continuous illumination, has been found to potentially induce early sexual development, even precocious puberty (27, 28) Furthermore, screen exposure contributes to heightened exposure to blue light. Research conducted on rats, has demonstrated that exposure to blue light can result in precocious puberty in male rats (29). Some scholars argue that there has been an observed rise in cases of precocious puberty among females during the COVID-19 pandemic. The pandemic may be associated with an increased prevalence of electronic device usage during lockdown, which could potentially result in a decrease in melatonin levels, subsequently initiating endocrine alterations, and ultimately causing the early onset of puberty (30).

Obesity acts as a mediator between screen exposure or sedentary behavior and early puberty. Cross-sectional investigations have indicated that exceeding 2 h of daily screen time poses a risk for overweight or obesity among preschool-aged children (31). Furthermore, longitudinal studies have suggested that prolonged television viewing during childhood is positively correlated with an increased likelihood of being overweight or obese in adulthood (32). Numerous potential mechanisms have been proposed to elucidate the relationship between screen exposure and obesity, with the consumption of food while viewing electronic devices and exposure to food advertisements playing a significant role (33). Additionally, sleep deficiency has emerged as another plausible factor contributing to the impact of screen exposure on obesity. Prolonged screen time has been found to disrupt sleep patterns, leading to delayed sleep onset, reduced sleep duration, and compromised sleep quality, characterized by slow sleep, early awakening, and disturbances in sleep rhythm (34). A comprehensive systematic review investigating the association between screen time and sleep revealed a consistent positive correlation between screen time and impaired sleep quality (13). Sleep deprivation has been shown to have an impact on food intake and total energy expenditure, potentially leading to weight gain (35, 36). The substitution of sedentary behavior for physical activity may also account for the relationship between screen exposure and obesity. Engaging in physical activity during childhood and adolescence has been found to decrease the prevalence of overweight and obesity (37). Research indicates that there is a correlation between obesity or higher body mass index (BMI) and early puberty in childhood (38). In model 1, unadjusting for any confounding variables, correlation was observed between early sexual maturation or early development and sedentary behavior in girls. However, when the variable of BMI was introduced in Model 2, the association disappeared. Previous discussions have suggested that obesity plays an important mediating role between screen exposure or sedentary behavior and early puberty or precocious puberty (31, 33, 35, 38). Therefore, we speculate that the association originally present in model 1 was weakened due to the inclusion of BMI. In model 3 and model 4, in addition to BMI, we also included MVPA and sleep duration. As mentioned above, there is a certain connection between physical activity, sleep duration, and obesity, so the correlation between precocious puberty or early puberty and sedentary behavior is further weakened in model 3 and model 4.

Furthermore, studies have provided confirmation that there is an interaction between biological maturation and social environmental factors (39), in other words, children may be influenced by what they see or are experiencing, leading to early puberty. Early exposure of girls to sexual information and images prematurely through activities such as watching romantic dramas and browsing sexual content, may also play a role in the development of early puberty due to screen exposure.

### 4.3 Research significance, future development and application prospects

Notably, the prevalence of precocious puberty has witnessed a significant rise during the COVID-19 pandemic, potentially attributed to environmental alterations such as escalated utilization of electronic devices, sedentary lifestyles, and reduced physical activity (30, 40). It is reasonable to speculate that during particular periods, such as pandemic of COVID-19, the association between screen exposure or sedentary behavior and precocious puberty may be strengthened. This investigation further substantiates the association between screen exposure or sedentary behavior and precocious puberty, providing theoretical support for larger, more in-depth research and potential guidance for parents.

## 5 Strengths and limitations

Initially, it is important to note that this study represents a novel investigation into the correlation between screen exposure, sedentary behavior, and precocious puberty. Furthermore, the evaluation of children's Tanner staging was conducted by expert pediatricians, thereby ensuring the reliability and precision of the study's findings. In this study, screen exposure encompassed various screen-based activities, including the use of contemporary screen devices such as computers, game consoles, smartphones, and tablets. However, it is important to acknowledge certain limitations in this study. Firstly, the reported screen exposure time and sedentary time relied on parental self-reports, which may introduce potential reporting bias. Secondly, due to the cross-sectional design of the study, it is not possible to establish a causal relationship between screen exposure, sedentary behavior, and precocious puberty.

## 6 Conclusion

This study provides evidence suggesting that excessive sedentary behaviors are correlated with an elevated risk of precocious puberty, particularly in girls. However, further longitudinal investigations are required to establish a causal relationship between screen exposure, sedentary behavior, and precocious puberty, as well as to delve into the underlying mechanisms.

## Data Availability

The raw data supporting the conclusions of this article will be made available by the authors, without undue reservation.
